# Community review: a robust and scalable selection system for resource allocation within open science and innovation communities

**DOI:** 10.12688/f1000research.125886.1

**Published:** 2022-12-06

**Authors:** Chris L.B. Graham, Thomas E. Landrain, Amber Vjestica, Camille Masselot, Elliot Lawton, Leo Blondel, Luca Haenal, Bastian Greshake Tzovaras, Marc Santolini

**Affiliations:** 1 Just One Giant Lab, Paris, France; 2 University of Warwick, Coventry, UK; 3 University of Nottingham, Nottingham, UK; 4 Learning Planet Institute, Université de Paris, Paris, France

**Keywords:** community science, decision making

## Abstract

Resource allocation is essential to selection and implementation of innovative projects in science and technology. Current “winner-take-all” models for grant applications require significant researcher time in writing extensive project proposals, and rely on the availability of a few time-saturated volunteer experts. Such processes usually carry over several months, resulting in high effective costs compared to expected benefits. We devised an agile “community review” system to allocate micro-grants for the fast prototyping of innovative solutions. Here we describe and evaluate the implementation of this community review across 147 projects from the “Just One Giant Lab’s OpenCOVID19 initiative” and “Helpful Engineering” open research communities. The community review process uses granular review forms and requires the participation of grant applicants in the review process. Within a year, we organised 7 rounds of review, resulting in 614 reviews from 201 reviewers, and the attribution of 48 micro-grants of up to 4,000 euros. The system is fast, with a median process duration of 10 days, scalable, with a median of 4 reviewers per project independent of the total number of projects, and fair, with project rankings highly preserved after the synthetic removal of reviewers. Regarding potential bias introduced by involving applicants in the process, we find that review scores from both applicants and non-applicants have a similar correlation of r=0.28 with other reviews within a project, matching traditional approaches. Finally, we find that the ability of projects to apply to several rounds allows to foster the further implementation of successful early prototypes, as well as provide a pathway to constructively improve an initially failing proposal in an agile manner. Overall, this study quantitatively highlights the benefits of a frugal, community review system acting as a due diligence for rapid and agile resource allocation in open research and innovation programs, with implications for decentralised communities.

## Introduction

The distribution of scientific funding through grants requires the identification of novel, feasible and potentially impactful projects. However, the traditional scientific grant allocation system involving a closed panel of experts in the field, or in similar fields, ^1^ is notoriously slow, ^2^ time consuming and expensive, often taking months and occurring in timescales of yearly rounds or grant calls. In extreme cases, the grant review program can be more costly than simply allocating small grants to each applicant, as in the case of the NSERC grant system of 2008. ^3^ In addition, the allocation of grants has shown to suffer from various biases, such as the composition of the grant panel, ^4^ gender and geographical location, ^5^ group based dynamics personality triumphing over other qualitative factors, ^6^^–^^8^ and socio-psychological factors such as group dynamics and personality traits triumphing over other qualitative factors. ^8^^,^^9^ Overall, selection results are only weakly predictive of future performance. ^10^

Often, the reason to conduct grant allocations in a ‘closed’ setting is to protect the intellectual property of the grant applicants. As a result, the majority of unsuccessful grant applications, which contain a large amount of research effort, are inevitably lost, unavailable to the public after the fact. ^11^ The recent emergence of the open science movement ^12^^–^^14^ has reversed this incentive, with open access practices and early sharing of results such as pre-registration now becoming normalised by institutions and journals. ^15^

Beyond the allocation of funding, the review of early-stage, unpublished work by community peers has been leveraged to allocate other types of resources. For example, conferences often need to allocate time for their participants to showcase their work to other members of the community during a usually short amount of time, thereby providing a platform for promoting the work, building novel collaborations, and getting feedback to improve a manuscript. In such cases, peer reviewing is needed to decide in a collegial fashion whether a work is worth a full oral presentation, a shorter lightning talk, a poster, or is not of a high enough standard to be showcased to participants. For example, the EasyChair online platform has been used by close to 100k conferences for handling such review processes. ^16^ Often, participants to a conference are also part of the “program committee” reviewing the proposed abstracts and papers of peer applicants, alongside external members of the scientific community. This allows for a rapid process usually lasting less than a few weeks.

**Figure 1.**
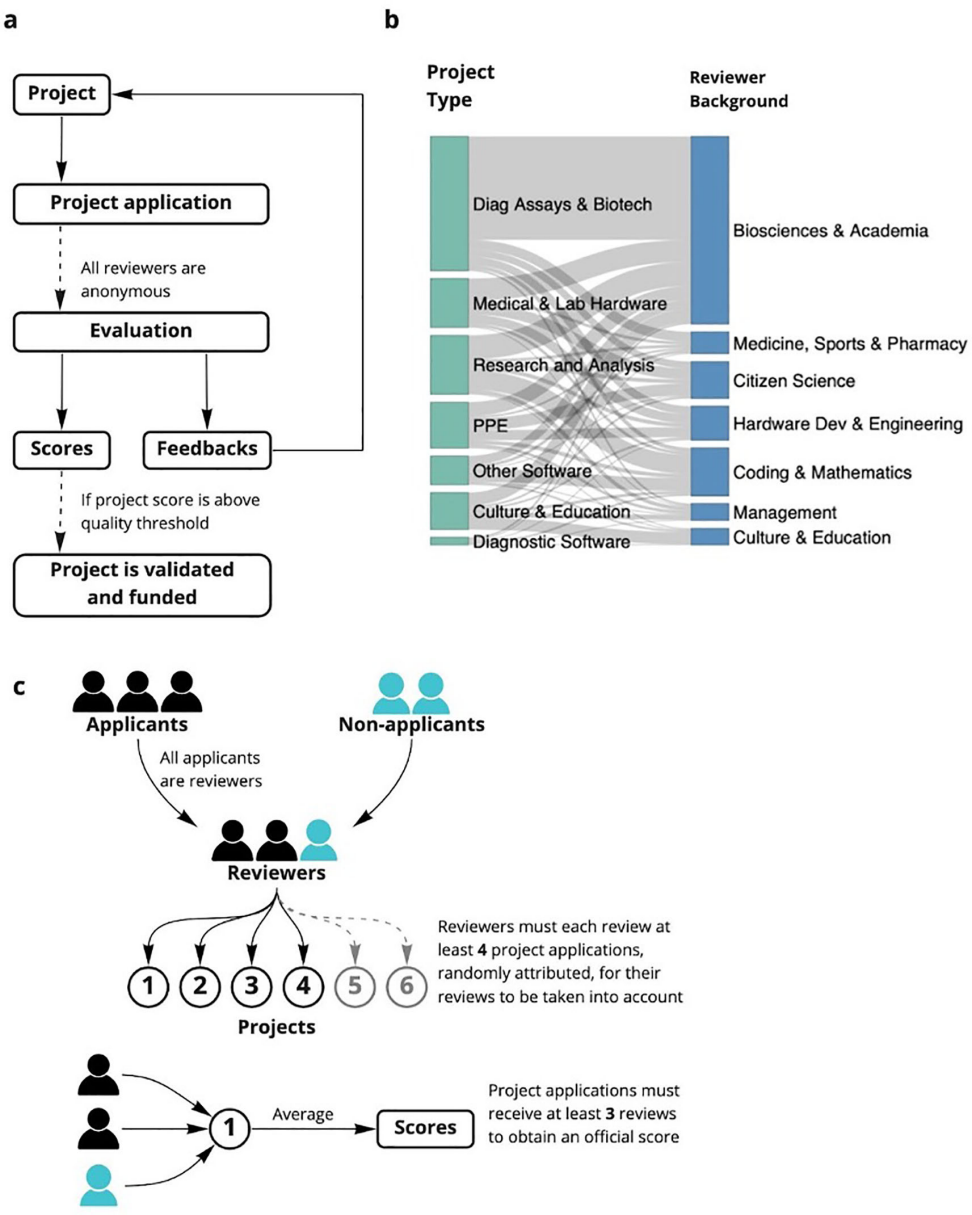
Overview of the open peer review process. (a) Stages of the open peer review process JOGL rounds 3-5. The online review forms and templates are found in supplementary data. (b) community review method JOGL rounds 3-5 (c) distribution of project type to expertise across rounds.

This suggests there is a potential for a new, more agile route for community-driven grant allocation bypassing pre-selected grant panels that handle funds and introduce barriers, ^8^ and relying instead on peer applicants to handle a large-scale application process in a short timescale. In this study, we present the design, implementation, and results of a community-driven, open peer-review system to support two open research communities during the coronavirus disease 2019 (COVID-19) pandemic across seven selection rounds ( Figure 1): the “OpenCOVID19” initiative from Just One Giant Lab (JOGL) ^14^^,^^17^ and the COVID relief charity Helpful Engineering. ^18^ We show that this system is robust (unaffected by reviewer removal), agile (fast timeline), iterative (covering multiple grant rounds), decentralised (driven by the community), and scalable. Finally, we discuss these results and the perspectives they offer for the design of future community-driven review systems.

## Methods

### Context

**Figure 2.**
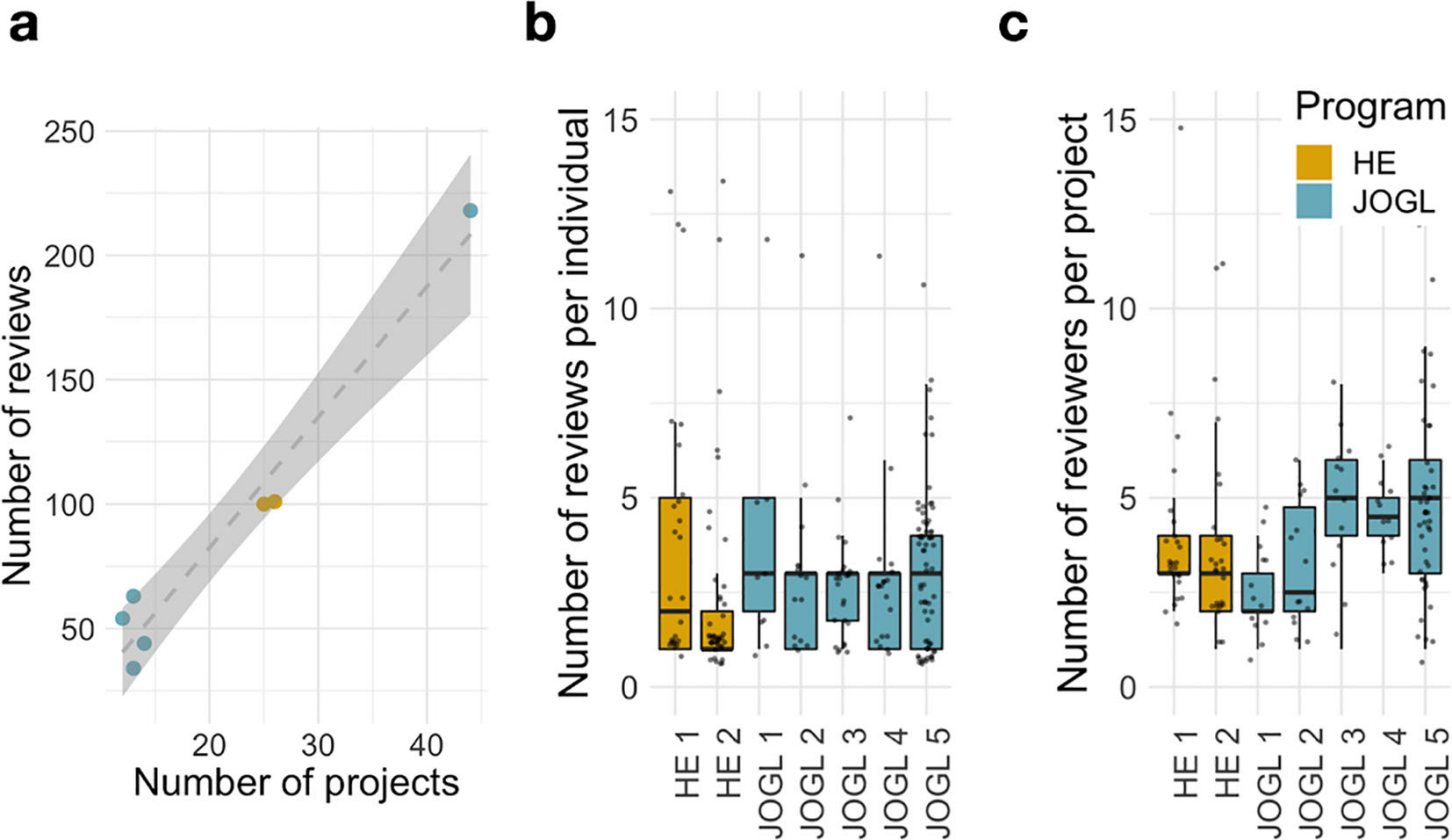
Scalability of the community review methodology. (a) Number of Reviewers and projects during each round of peer/grant review. HE- Helpful Engineering Crowd reviews, JOGL- Just One Giant Lab funded projects. (b) Number of reviews per individual reviewer. (c) Number of reviewers per project. Despite a scale-up in the number of projects, the number of reviews per round scales linearly with the number of projects applying.

The implementation of a crowd-based, open peer-review system followed the need to support two nascent community efforts, first by allocating volunteers to projects in the COVID relief charity Helpful Engineering, ^18^ then by allocating funding to projects in the JOGL “OpenCOVID19” initiative. ^17^ The method was developed as an open access grant review and funding allocation system, meaning that it was open to anyone willing to review. It was implemented using the Just One Giant Lab platform (app.jogl.io) as the project proposal host, and free-to-use tools and forms to conduct the review process (Extended Data:FigS2 ^19^). The implementation was applied and refined over 7 rounds across 1 year.

### General process of review

The peer review system was conducted on early phase projects within both JOGL and Helpful Engineering. These projects were submitted by project leaders to a grant review process in order to allocate volunteers in the case of Helpful Engineering, and funding in the context of OpenCovid19. Reviews of these projects (see Figure 1b) were initially conducted by members of the community and included members of other projects who also submitted their project for review.

As a consequence of the process being experimental and serving an urgent need, the process was altered over time. However, it followed the same general pattern ( Figure 1, Extended Data:FigS1 ^19^). First, a template for the grant proposal was created by the community and was iteratively edited (Extended Data ^19^) template followed typical grant application templates, ^20^ with sections on team composition, the project general hypothesis and its timeline. The proposal was then submitted using a google form, which requested an email address and required only one application per project (Extended Data:FigS2a ^19^). In Helpful Engineering rounds this included a link to their proposal hosted in editable google documents, while in JOGL rounds this included instead a link to their open access JOGL page proposal. The project links were manually formatted into a google sheet with a link to a review form for convenience, along with descriptions of desirable reviewer skills by the applicants in the proposal submission form to help reviewers find relevant projects (Extended Data: FigS2B ^19^). A technical evaluation form scoring various criteria (eg: proposal efficacy, team composition, impact) on a scale from 1-5 (Extended Data ^19^) was created by the designers of the program and iteratively changed following feedback from the community (Extended Data:FigS2c ^19^). This form separated questions on projects into two areas centred around Impact and Feasibility for ease of identifying the problems and/or strengths in their grant application. A message with a link to the reviewer form for use in review, along with a nested google sheet containing project proposal links was spread among the community through announcements and email. In later rounds (JOGL 3-5) all applicants were asked to review at least three other projects and the process was randomised, removing the need for a sheet. The review process was given between 4 days HE 1, 8 days HE 2, 7 Days - JOGL 1, 10 days - JOGL 2, 16 days - JOGL 3, 21 days - JOGL 4 and 28 days JOGL 5, (Extended Data:FigS1b ^19^) to allow reviews to occur and be collected via a google form into a google sheet automatically (Extended Data:FigS2d ^19^). No reviewer selection was performed, however usernames (Slack handles or JOGL user names depending on the round) and emails were collected for conducting further analyses. The average reviewer scores were then composed into a presentation to the community, and those projects with a score above a given impact/feasibility threshold (Extended Data:FigS2e ^19^) were chosen for grant funding. Due to the community aspect of our study, members from the JOGL HQ participated in the process, and their removal from the analysis does not change the observations (Extended Data:FigS10 ^19^), we therefore retain these in our analysis.

### Iterative changes to the review process

As mentioned in the previous section, the method of review was iteratively changed throughout the programme, elongating from an initial “emergency style” four day period of review and allocation (HE round 1) to 21 and 28 days in JOGL rounds 4 and 5 as the need for rapid response reduced, with an overall median average of 10 days per round (Extended Data:FigS1b ^19^). As such, the design of the general process described in Figure 1 and Extended Data:FigS1 ^19^ had some variations. For example, initially applicants were not required to review applications ( Figure 1b). Upon scaling up of the programme, the process was adapted to be less dependent on volunteer reviewers, (Extended Data:Fig S1b,A-D ^19^) and more dependent on the applicant’s reviews of their competing peers ( Figure 1c). In JOGL rounds 3, 4 and 5 (Extended Data:FigS1b ^19^) teams depositing a proposal could only be eligible after having reviewed at least three other teams. The changes in the process and differences in the rounds are summarised in Extended Data:FigS1c. ^19^ The major changes between Helpful Engineering (HE) and JOGL rounds (Extended Data:FigS1c ^19^) occurred through changes in the nature of proposal submission from google document links to an online project repository. In addition, HE rounds offered no grants, but instead publicity and allocation of members to projects, while JOGL offered microgrants worth up to 4000 euros per team (Extended Data:FigS2c ^19^).

### Final selection process

In Helpful Engineering, this review method allowed 54 projects to be reviewed and ranked by score for community recruitment purposes, with no official threshold, but instead an arbitrary set of “Highlighted projects”. Within JOGL this grant system reviewed 96 eligible applications ( Figure 2) and allocated requested funds to 36 of these. Once the review process had taken place, the cut-off threshold of scores given by reviewers to projects for funding by JOGL was decided by an absolute threshold (above 3.5/5 average reviewed score) rather than a rejection rate. The absolute 3.5/5 threshold was chosen due to the gap in project scores in the first JOGL round, and maintained at this standard for consistency. Those with a score above the threshold were funded.

### Detection of fraudulent reviewer behaviour

The results of each round, and number of reviews per reviewer were closely monitored through simple email handle tracking by a data handling administrator. If a number of emails were found to be grading a particular project and not others this was suggestive of fraudulent behaviour and self-grading. These reviews were then removed, and teams that were found responsible for this bad behaviour were removed from the review process, as described in grant round participation rules. This was performed only one time across all rounds prior to the rule of each reviewer having a minimum review count for their scores to be counted, which was created after this event.

### Computation of inter-review correlations

In order to compute the correlation between reviews within a project, we first proceeded with data cleaning. Indeed, in several rounds, reviewers had to answer only a subset of questions from the review form that corresponded to the topic of the project (e.g. data project vs bio project). However, in some cases, projects were assigned to one or the other category by the different reviewers, leading them to answer to different sets of questions, making the correlation only partial. To mitigate this effect, for each project we kept only the reviews that corresponded to the choice of topic that was most expressed among reviewers. If no majority could be found, the project was removed from analysis. We then converted review scores into vectors of length the number of grades in the form. A Spearman’s rho correlation was then computed between all pairs of reviews within a project. Finally, for each review we computed the average correlation with the other reviews in the project. This number was then associated with the features of the reviewer who produced the review ( Figure 4 and Extended Data:FigS7 ^19^).

### Reviewer feasibility and impact scores

For JOGL rounds 1-5, we categorised the 23 to 29 questions from the review forms into either impact or feasibility related questions (see Underlying Data Review forms). The feasibility and impact categories were used to provide two dimensional projections of project scores during the result presentation.

### Reviewer professions and project types

For all JOGL rounds, reviewer responses of the "What is your expertise relevant to this project" question were manually coded into simple categories per review (see Table S1 in the Extended Data ^19^). This data was then used as a proxy for expertise distribution across rounds ( Figure 1b).

In addition, reviewer responses to the "Which category would you say the project falls under?" question were manually coded into a set of simple categories, representing a summary of the project types across rounds per review (see Extended Data conversion table ^21^). The data, due to suggested categories provided by the form, needed little manual coding, but was formatted into a list, then concatenated into similar project types for simplicity. This data was used to assess project type distribution across rounds ( Figure 1b).

### Bootstrap analysis

**Figure 3.**
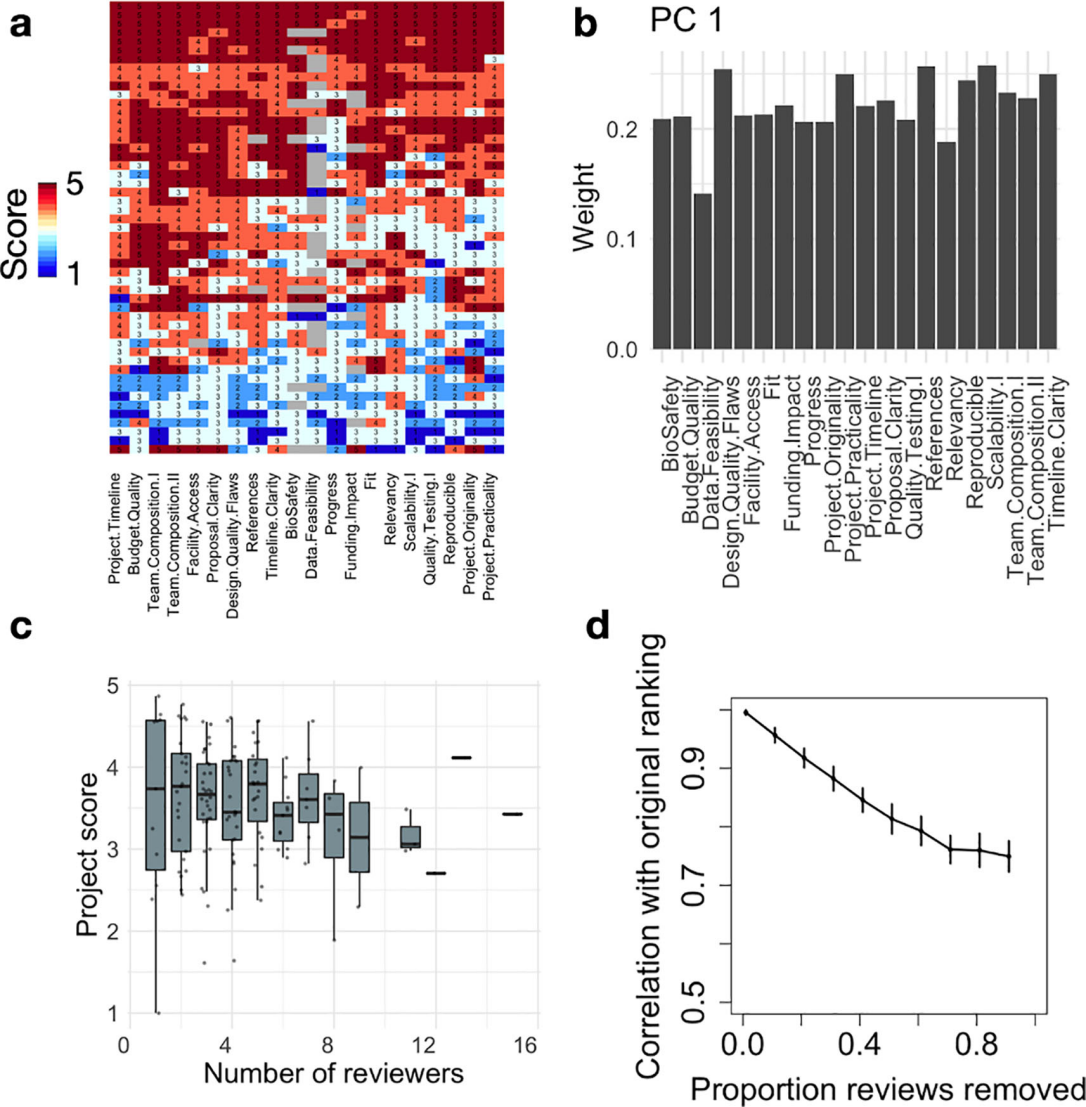
Robustness of the Community review process. (a) Heatmap showing review scores (rows) across questions (columns) for the JOGL round 4. Row and column clustering was performed using correlation distance and average linkage. (b) We show for PC1 (53% variance) the weights of the questions from the original question space. PC1 has near uniform weights across dimensions, indicating that it corresponds to an average score. (c) Project average score across reviewers as a function of number of reviewers. (d) Bootstrap analysis showing the Spearman correlation between the final project ranking and simulated project rankings with increasing proportion of reviews removed from the analysis (see Methods).

In order to perform the bootstrap analysis of Figure 3d, we first ranked all projects using their average review score across reviewers. We then selected a review at random. If the corresponding project had at least another review, we removed the selected review and recomputed the average scores and final ranking. We then computed the Spearman correlation between the obtained scores and the original scores. This process was repeated until each project had only one review. Finally, we reiterated this analysis 50 times. The analysis code can be found as Extended Data. ^19^

### Ethics/Consent

We confirm all ethical guidelines have been followed, using the same ethical procedures described in Commission Nationale de l'Informatique et des Libertes registration number “2221728” for user research and “2227764” for grant administration. Consent was granted by a user agreement on JOGL’s website upon signup ( https://app.jogl.io/data), and on the google forms used during the study.

## Results

### Scalability of the review process

We describe in Figure 2 the reviewing activity across the seven rounds implemented. Despite the large differences in number of projects between rounds, we find that the number of reviews per round scales linearly with the number of projects applying ( Figure 2a). In addition, the number of reviews per individual and number of reviewers per project have relatively stable distributions across rounds, independent of scale ( Figure 2b-a). For example, despite the substantial growth in reviewers and projects in JOGL round 5, we find that the distributions of number of reviews per reviewer and number of reviewers per project are comparable to those observed in the previous rounds, highlighting the scalability of this review system to different systems. Finally, we note that the number of reviewers per project show a sustained increase from JOGL round 3 onwards, corresponding to the change in review process, where applicants were required to review at least 3 other projects (see Methods). This highlights the benefits of this requirement in promoting sustained engagement.

### Robustness of the final project ranking

In order to obtain a granular score for each project, the reviewers had to grade between 23 (JOGL 1-2) and 29 (JOGL 3-5) criteria in the review form. ^21^ We first investigate whether these questions would cover different dimensions of project quality. We show in Figure 3a a heatmap of reviewer scores in JOGL round 4 across 20 questions (removing questions only representing a minority of projects), visually showing a greater inter-review variability (rows) than inter-questions variability (columns). As such, respondents seem to assign a project with either low scores or high scores throughout their review. To quantify the number of dimensions of variation across grades, we conduct a Principal Component Analysis (PCA) on the questions correlation matrix, i.e correlations between pairs of questions across reviews (see Extended Data:Fig S2a ^19^). We find that the first principal component (PC1) explains most of the variance (53%), with the next largest PC explaining less than 6% of the variance (Extended Data:Fig S3 ^19^). When examining the weights of the various questions in PC1, we find that they all contribute to a similar level ( Fig 3b), meaning that the PC1 is close to the average over all questions, confirming the visual insight from Fig 3a. This shows that scores are highly correlated, and that the average score across the review form is a reasonable operationalisation of project quality. In addition, we find that the top 10 PCs explain ~90% of the variance, indicating that review forms could be reduced in complexity using only half of the number of questions to obtain a similar outcome.

**Figure 4.**
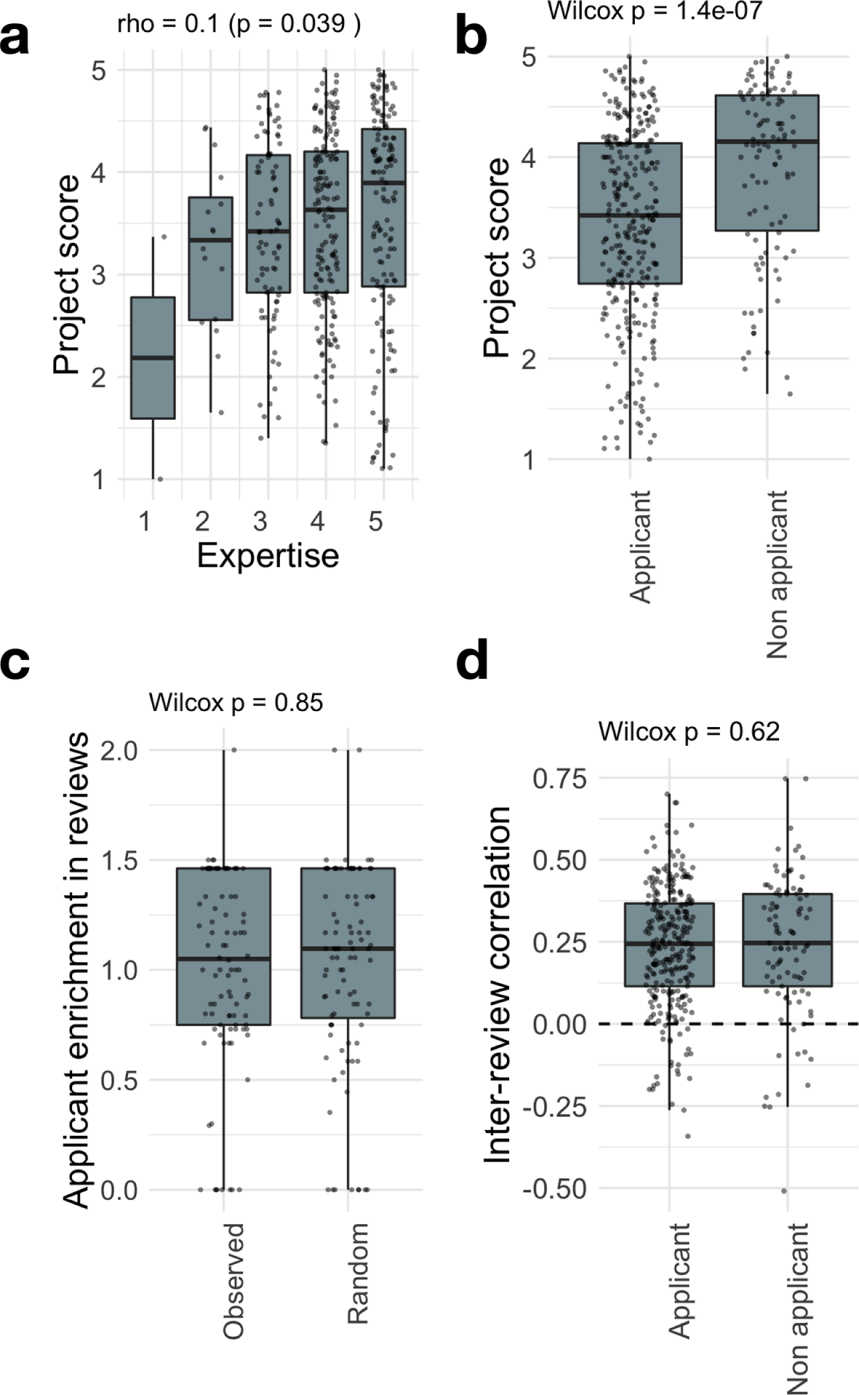
Questionnaire granularity allows to measure and mitigate reviewer biases. Breakdown of project score as a function of (a) self-assessed expertise, (b) applicant status (i.e. the reviewer is also an applicant in the round). See Fig S4 for a breakdown by review round. (c) For each project, we compute the ratio between the proportion of applicant reviewers to the average proportion of applicant reviewers observed in the round. The boxplot compares the computed enrichments to the ones obtained for randomly assigned reviewers to projects, showing that applicants are evenly distributed across projects. (d) For each project, we compute the ratio between the proportion of applicant reviewers to the average proportion of applicant reviewers observed in the round. The boxplot compares the computed enrichments to the ones obtained for randomly assigned reviewers to projects, showing that applicants are evenly distributed across projects.

We next investigate the reliability of the review scores obtained across reviewers. As suggested by the previous section, for each review we compute the average score across all criteria from the review form. In the following, we refer to this average score as the review score. We observe a generally good discrimination of review scores between projects, with intra-project variation smaller than inter-project variation (Extended Data:FigS4 ^19^).

**Figure 5.**
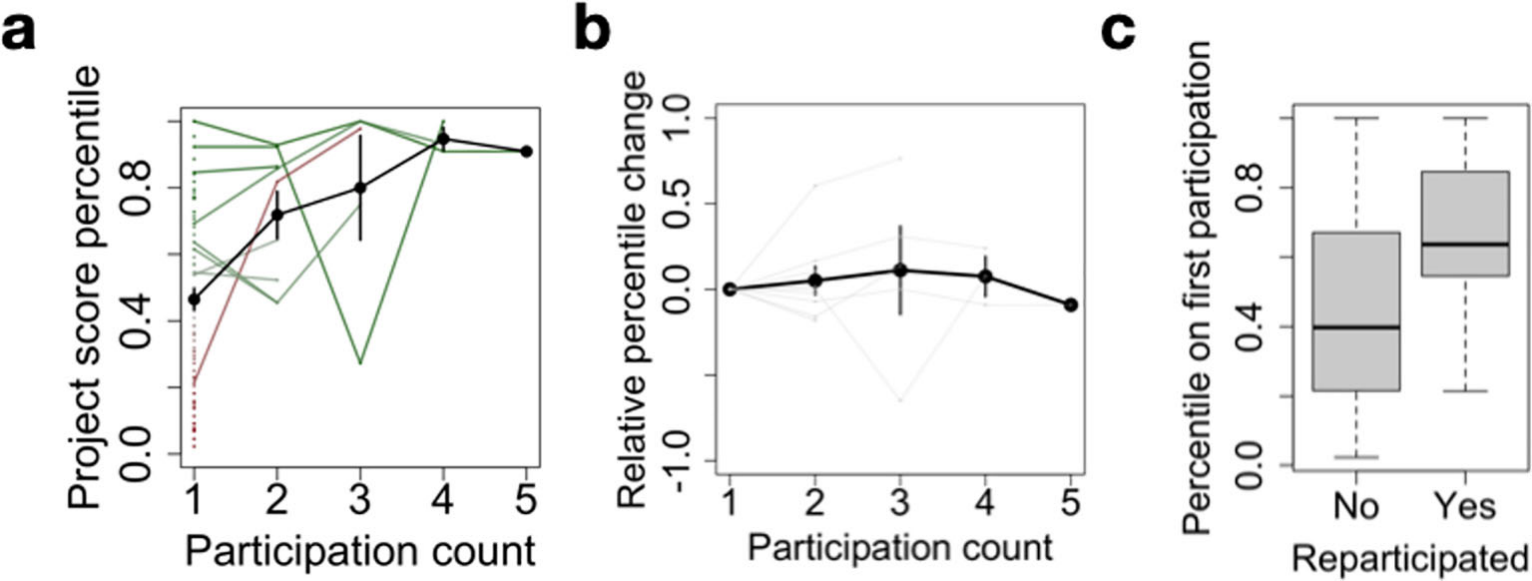
Multiple participations foster long-term project sustainability. (a) Project score percentile as a function of participation count. For each project, a score percentile is computed to quantify their relative rank within a specific application round, allowing to compare multiple projects across rounds. Participation count refers to the successive number of rounds a project has applied to. The black line denotes the average across projects, error bars represent standard error. Dots correspond to projects with only one participation, and lines to re-participating projects. Finally, the color gradient indicates relative score at first participation, from red (low) to green (high). (b) Same as a., after subtracting the percentile at first participation. (c) Score percentile at first participation as a function of whether or not a project has re-participated.

Finally, we investigate the robustness of the final project ranking as a function of the number of reviews performed using a bootstrap analysis (see Methods). For each project, a project score is computed by averaging its review scores, and projects are then ranked by decreasing score. We show in Figure 3d the Spearman correlation between the original project ranking and the ranking obtained when removing a certain proportion of reviews. We find that even with only one review per project, the final ranking is strongly conserved (rho=0.75 and see Extended Data:FigS5 ^19^), confirming that intra-project variability is much smaller than the range of inter-project variability. This supports our design strategy, showing that the use of a granular form allows us to differentiate between projects whilst minimising the impact of individual reviewers’ variability.

### Measuring reviewer biases

The previous results show the existence of variability between reviews from different reviewers, yet with limited impact on final rankings ( Figure 3d). Here we investigate the source of review variability: is it due to inherent grading variability between individuals, or can it be attributed to other factors? To evaluate this question, we analyse how review score varies with reviewer attributes. We explore in particular two possible sources of bias for which we could gather data: expertise and application status. First, reviewer expertise might be important in determining an accurate project score. This feature is operationalised using the self-reported expertise grade (1 to 5) present in the review forms of JOGL rounds. Second, a majority of reviewers (65%) were applicants of other competing projects, which could lead to a negative bias when reviewing other competing projects.

We show in Figure 4 how the review score varies as a function of these reviewer characteristics. We find that review score increases slightly with expertise ( Figure 4a, Spearman’s rho=0.1, p=0.039). However, the strongest effect is found when looking at applicant bias: review scores from applicants are significantly lower than those from non-applicants ( Figure 4b, p=1.4e-7). Given the fact that in JOGL rounds 3-5, applicants were required to score at least 3 projects, they are found to have a lower expertise towards other projects (Extended Data:Fig S6 ^19^), which could explain the lower scores as suggested by Figure 4a. Yet, when controlling for review expertise, we find that application status is the main contributing factor, with a score difference between applicants and non-applicants of -0.52 points (p=1.61e-6, Extended Data:Supplementary Table 1 ^19^). This supports that application status is a significant source of bias in the final score.

Such differences could be due to unfair grading, with reviewers from a certain category (applicants or non-applicants) grading more “randomly” than others. To analyse this effect, we need to look beyond average score into correlations. Indeed, two similar average scores could stem from highly different fine-grain score vectors. Imagine two reviewers grading 3 questions from 1 to 5. The first reviewer gives the grades 1, 2, and 5, while the second gives 5, 1, and 2. These reviews produce the same average score (2.67). However, their fine-grain structure is anti-correlated, with a Pearson correlation r = -0.69. In our context, we find that review scores are positively correlated, with a median Pearson correlation between their reviews of r = 0.28 across rounds ( Figure 4d), in line with previous observations in traditional funding schemes [35]. More importantly, we find no difference between applicants and non-applicants in their correlation with other project reviews ( Figure 4c). This indicates that the variability between grades within a review form are conserved across reviewer characteristics (see Fig S7 and Extended Data:Fig S9 for the other characteristics ^19^). As such, if applicants are uniformly distributed across projects, one will not expect a difference in the final rankings.

### A framework for iterative project implementations

In the JOGL implementation of the community review system, projects can apply to any number of rounds, irrespective of whether or not they have already successfully obtained funding in a previous round. We found 9 projects that applied to multiple rounds. On average, the relative performance of the projects in a grant round increases as a function of the number of participations ( Figure 5a). We find that this effect is explained by re-participation being associated with early success, with initially lower performing projects eventually dropping out ( Figure 5b-c). As such, the multiple round scheme supports projects with a high initial potential in the long-term through repeated micro-funding allocations. We also note that in the case of 2 projects, re-participation after an initial failure allowed them to pass the acceptance threshold. This highlights how constructive feedback allows for a rapid improvement of a project and its successful re-application in the process.

## Discussion

In this manuscript we describe the “community review” method for the identification of novel, feasible and potentially impactful projects within two communities of open innovation: Helpful Engineering and OpenCovid19. This process was leveraged for the attribution of volunteers as well as micro-grants to projects over a year, in an agile and iterative framework.

Key to the system is the requirement of applicants to take part in the reviewing process, ensuring its scalability. As such, the number of reviews is proportional to the number of projects applying ( Figure 2), with a fast median process duration of 10 days. This requirement comes at a risk, since applicants might be negatively biased towards other projects they are competing against. Accordingly, we found that applicants consistently give a lower score to projects when compared to non-applicants (-0.52 points). This bias cannot be explained solely by the lower expertise of applicants towards the randomly assigned projects. Indeed, we found that self-reported expertise has only a limited impact on the final score ( Figure 4c). The effect is most stringent for rare cases of self-reported expertise of 1 and 2 out of 5, suggesting that a threshold of 3 might be implemented to remove non-expert bias. It is on the other hand possible that non-applicants are positively biased towards projects from which they might have personally been invited to review. We however noted no such report in the conflict of interest question in the review form.

Despite these biases, we found that applicants and non-applicants have a similar behaviour when grading questions in the form, with a stable Pearson correlation between their reviews of r = 0.28 ( Figure 4/Extended Data:Fig S8 ^19^). This is slightly higher than the correlation of 0.2 observed in an analysis of the ESRC’s existing peer review metrics, ^22^ suggesting comparable outcomes when compared to existing institutional methods. The similarity of their correlation profiles means that such biases contribute a similar “noise” to the system: they might change the overall average scores, but not their ranking as long as applicants are well distributed across projects. Accordingly, we found that the community review system is robust to the removal of reviewers, with an average ranking Spearman correlation of 0.7 in the extreme case of one reviewer per project.

Finally, we showed that some projects apply multiple times to the application rounds. While the number of such projects of this type is small (9 projects), we find that it had two benefits. First, we found two projects that re-applied after an unsuccessful application, allowing them to pass the acceptance threshold on the second application. This showcases the ability of the feedback system to benefit projects in constructively improving their application. Furthermore, we found that the number of applications of a project is strongly dependent on its performance on the first application. This means that the iterative process allows to select highly promising projects and sustain their implementation in the mid- to long-term. This is of particular importance when considering traditional hackathon systems, where promising projects are usually not supported over longer periods of time.

The speed and cost-efficiency of the community review process has allowed for a reactive response to the high-pressure environment created by the pandemic. This agility has meant that within the short time frame given, projects have been able to produce literature, methods and hardware and put them to use. ^23^^–^^28^ Overall, the community review system allows for a rapid, agile, iterative, distributed and scalable review process for volunteer action and micro-grant attribution. It is particularly suited for open research and innovation communities collaborating in a decentralized manner and looking for ways to distribute common resources fairly and swiftly. Finally, community review offers a robust alternative to institutional frameworks for building trust within a network and paves the way for the installation of community-driven decentralized laboratories.

## Data Availability

Open Science Framework: DATA FOR: Community review: a robust and scalable selection system for resource allocation within open science and innovation communities. https://doi.org/10.17605/OSF.IO/CAZ4N. ^21^ This project contains the following underlying data:
- Review Data (the raw responses of the review rounds analysed by the paper, and the raw data used in the study.) - Project round progress.csv (aggregated data and is based on data post analysis for our final figure, and the scores of each project over time, however we have aggregated this for ease of viewing.) - Grant Review forms (the forms used to assess each proposal) - Peer Review protocol (the protocol used to analyse the raw data, giving the correlation values we refer to in the paper) - Coded expertise (the simplified version of project and reviewer type collected during review) *Open Science Framework: EXTENDED DATA FOR: Community review:* a robust and scalable selection system for resource allocation within open science and innovation communities, https://doi.org/10.17605/OSF.IO/W5Q9B. ^19^ This project contains the following extended data:
- Supplementary figures 1-10 - Supplementary table 1 - Analysis code Data are available under the terms of the Creative Commons Attribution 4.0 International license (CC-BY 4.0).
