# Supplementary material for: Community review: a robust and scalable selection system for resource allocation within open science and innovation communities
Source: F1000Res. 2023 Apr 18;11:1440. Originally published 2022 Dec 6. [Version 2] doi: 10.12688/f1000research.125886.2 (PMC10818098; doi:10.12688/f1000research.125886.2)
Supplement: Peer review history

# Reviewer report: Reviewer response for version 2

**DOI:** 10.5256/f1000research.145384.r185935

**Published:** 2024-01-26

**Authors:** Dimitrios Kouis

**Affiliations:** 1 University of West Attica, Athens, Greece

The article presents an interesting topic related to a different model/method of evaluating proposals for funding based on applying the distributed peer review method with specific variations and assumptions. Implementing the proposed model is suggested as an alternative approach to the traditional way of evaluating funding proposals, meaning the evaluation through peer review by a panel of experts. This particular topic increasingly engages the research community (indicative articles 1,2,3) in selecting proposals for funding and evaluating scientific publications and other aspects of research, mainly within the context of Open Science.

 In terms of the current article, both the proposed evaluation flow and the workflow adaptations that were applied and tested at various stages and subsequently evaluated with an in-depth statistical analysis give the article the necessary elements of innovation and originality to be accepted for publication. Specifically, the methodological approach, the assumptions made, the data collected, and the further analysis and conclusions drawn are based on a correct and logical scientific approach and present great interest.

 If there are any points of critique and thought for a potential future research effort (and not so much regarding corrections to the current article), these are summarized in the following two points:

- To what extent can the scientific field of application for evaluating the proposed methodology affect the results? Will applying the proposed method to a different scientific area provide the same results? (As expected, different scientific topic means other evaluators' mentality, evaluation forms etc.)
- To what extent can the proposed methodology be adopted by a governmental organization or an international funder, given that the evaluators might also be applicants for getting funded and thus exhibit an "expected behaviour of bias" and, as the authors observed in some cases, must be removed? Although such a process (i.e., the one proposed in the article) might ultimately yield better results (fairer evaluation), it may also be subjected to greater criticism due to the possible involvement of the participants themselves.
- Concerning the previous observation, which might be the weak point for adopting the proposed methodology on a broad scale, an alternative solution could be using the participants' reviews only to improve the quality of the proposals and not for grading purposes.

The article provides all the necessary elements, such as technical validity, methodological accuracy, results reproducibility, etc. It also opens the discussion within a correctly documented scientific framework for the revision/improvement of the evaluation framework for research proposals for funding in the context of Open Science.

Is the work clearly and accurately presented and does it cite the current literature?

Yes

If applicable, is the statistical analysis and its interpretation appropriate?

Yes

Are all the source data underlying the results available to ensure full reproducibility?

Yes

Is the study design appropriate and is the work technically sound?

Yes

Are the conclusions drawn adequately supported by the results?

Yes

Are sufficient details of methods and analysis provided to allow replication by others?

Yes

Reviewer Expertise:

Library Networks with an emphasis on shared bibliographic data management systems and collaborative systems for managing digital collections; Digital Content Management with an emphasis on electronic scientific publications; Scholarly communication, and specifically models of access and dissemination of scientific content, on research data as well as on the procedures of measurement and evaluation of research activity

I confirm that I have read this submission and believe that I have an appropriate level of expertise to confirm that it is of an acceptable scientific standard.

## References

[1] : Evaluation of research proposals by peer review panels: broader panels for broader assessments?. *Science and Public Policy* .2023; 10.1093/scipol/scad009 10.1093/scipol/scad009

[2] : Designing grant-review panels for better funding decisions: Lessons from an empirically calibrated simulation model. *Research Policy* .2022;51(4) : 10.1016/j.respol.2021.104467 10.1016/j.respol.2021.104467

[3] : The troubles with peer review for allocating research funding: Funders need to experiment with versions of peer review and decision-making. *EMBO Rep* .2019;20(12) : 10.15252/embr.201949472 e49472 10.15252/embr.201949472 31680417 PMC6893288

# Reviewer report: Reviewer response for version 2

**DOI:** 10.5256/f1000research.145384.r170051

**Published:** 2023-04-25

**Authors:** Ferdinando Patat

**Affiliations:** 1 European Southern Observatory, München, Germany

I am fully satisfied with the changes the authors have made following my recommendations.

Is the work clearly and accurately presented and does it cite the current literature?

Partly

If applicable, is the statistical analysis and its interpretation appropriate?

Yes

Are all the source data underlying the results available to ensure full reproducibility?

Yes

Is the study design appropriate and is the work technically sound?

Yes

Are the conclusions drawn adequately supported by the results?

Yes

Are sufficient details of methods and analysis provided to allow replication by others?

Yes

Reviewer Expertise:

Astrophysics: Supernovae. Peer-review: proposal selection and time allocation processes.

I confirm that I have read this submission and believe that I have an appropriate level of expertise to confirm that it is of an acceptable scientific standard.

# Reviewer report: Reviewer response for version 1

**DOI:** 10.5256/f1000research.138239.r161990

**Published:** 2023-02-17

**Authors:** Ferdinando Patat

**Affiliations:** 1 European Southern Observatory, München, Germany

The paper reports on the design, deployment and evaluation of what the authors describe as an 'agile community review system' for allocating micro-grants. As opposed to classical peer-review performed by pre-selected expert panels, the community review involves the participation of applicants themselves. It therefore provides as scalable solution, in the sense that the number of reviewers increases with the number of applications, hence keeping the review load manageable and the duty-cycle faster than in the classical panel case. The data show that the inter-reviewer correlation is similar to what has been reported for the classical paradigm. Also, the final rankings are shown to be robust against the randomised removal for reviewers.

 The paper presents the case very clearly and accurately. One aspect which is weak (honestly this is probably the only weakness of the paper) is that the casual reader is left under the impression that the idea of a community review is new and proposed here for the first time. And that is not the case. This concept, sometimes indicated as *distributed peer review* (DPR), has been around for many years. As far as I know the idea appeared first in 2009 in a paper by Saari & Merrifield (2009) ^1^. Although it was referring to applications for telescope time, the concept was of general interest. This mechanism has been implemented at least in three major, ground-based astronomical facilities (GEMINI, ALMA, ESO). Also, a similar distributed process has been deployed in the field of computer sciences for the selection of conference papers ^5^. Other interesting examples are those of the US National Science Foundation ^2^^,^^3^ and the US National Institute of Food and Agriculture (NIFA), which has deployed DPR in 2016 ^6^^,^^7^.

 The authors should therefore provide a short recap of these initiatives and cite the applicable references. In addition to the papers published by the above organisations, there is an article on Physics Today ^4^ which gives a nice summary for the specific cases in astronomy.

 Other minor points, which the authors should consider, are listed here:

1. Scalability. The authors correctly state that the approach is scalable, in the sense that increasing the number of projects linearly increases the number of reviewers. They show this in Fig. 2b. At a first read it looks like this is an unexpected results and, somehow, the data show that this is the case. However, I argue that this is completely expected, since the number of potential reviewers, on average, is directly proportional to the number of submitted projects. This would not be necessarily the case if the system would not force the applicants to review proposals. Maybe the authors should be more explicit on this.
2. In the section about fraudulent behaviour, the authors state that this was removed from the review process. However, they do not explain what this means in practice in terms of actions taken against those reviewers. Is their project simply rejected? Are they warned? Or is just their evaluation removed from the data?
3. The caption of Figure 4 for panels c) and d) does not match what is presented in the figure and also its description in the main text section *Measuring reviewer biases*:
  "For each project, we compute the ratio between the proportion of applicant reviewers to the average proportion of applicant reviewers observed in the round. The boxplot compares the computed enrichments to the ones obtained for randomly assigned reviewers to projects, showing that applicants are evenly distributed across projects. (d) For each project, we compute the ratio between the proportion of applicant reviewers to the average proportion of applicant reviewers observed in the round. The boxplot compares the computed enrichments to the ones obtained for randomly assigned reviewers to projects, showing that applicants are evenly distributed across projects."
  The authors never explain what they mean by "enrichment". Also, while panel c) may be showing a ratio, this is not the case for panel d), which on the y-axis has the label "inter-review correlation", which sounds like a Pearson correlation, and not a ratio. In general, I am rather confused about panels c) and d) in Fig. 4, which I reckon requires a better explanation also in the main text (which, by the way, never includes the word "enrichment").
4. As shown in Fig. 3a, there are two evaluation criteria related to the Team composition. Given that many organisations are now moving to a dual anonymisation of the applications, it would be interesting to see how the scores change if one removes these two indicators from the final score. I guess that, given the large number of indicators (and the results shown by the authors about the slight change when 50% of them are removed), no measurable effect is going to be seen. Also because, since the team's identity is known to the reviewers, there is certainly a cross-talk between this indicator and all others. It is probably therefore impossible to disentangle its effect from the available data. Nevertheless, I suggest the authors mention this aspect.

The study is well designed, the analysis was conducted in an appropriate way and included a satisfactory level of checks and controls. The methods are well explained and the necessary data are made available so that the analysis can be repeated and validated. The statistical analysis and its presentation meet the required standards.

 The conclusions are well supported, interesting and useful for other organisations which may consider adopting a similar schema. I therefore recommend the article for indexing after the point on the missing citations and short description of existing cases is addressed.

Is the work clearly and accurately presented and does it cite the current literature?

Partly

If applicable, is the statistical analysis and its interpretation appropriate?

Yes

Are all the source data underlying the results available to ensure full reproducibility?

Yes

Is the study design appropriate and is the work technically sound?

Yes

Are the conclusions drawn adequately supported by the results?

Yes

Are sufficient details of methods and analysis provided to allow replication by others?

Yes

Reviewer Expertise:

Astrophysics: Supernovae. Peer-review: proposal selection and time allocation processes.

I confirm that I have read this submission and believe that I have an appropriate level of expertise to confirm that it is of an acceptable scientific standard, however I have significant reservations, as outlined above.

## References

[1] : Telescope time without tears: a distributed approach to peer review. *Astronomy & Geophysics* .2009;50(4) : 10.1111/j.1468-4004.2009.50416.x 4.16-4.20 10.1111/j.1468-4004.2009.50416.x

[2] : Incentives, Quality, and Risks: A Look Into the NSF Proposal Review Pilot. *arXiv* .2013; doi.org/10.48550/arxiv.1307.6528 10.48550/arxiv.1307.6528 Reference source

[3] : A radical change in peer review. *Science* .2014;345(6194) : 10.1126/science.345.6194.248 248-249 10.1126/science.345.6194.248 25035465

[4] : Distributed peer review passes test for allocating telescope slots. *Physics Today* .2020; 10.1063/PT.6.2.20200513a 10.1063/PT.6.2.20200513a Reference source

[5] : Challenges, experiments, and computational solutions in peer review. *Communications of the ACM* .2022;65(6) : 10.1145/3528086 76-87 10.1145/3528086

[6] NIFA - FAQs for Distributed Peer Review (DPR). *NIFA* .2016; Reference source

[7] distributed peer-review (DPR). *NIFA* .2016; Reference source

# Response

**Published:** 2023-03-08

**Authors:** Chris Graham

**Affiliations:** University of Warwick, UK

Dear Ferdinando,

 Thank you for a thorough and well-rounded review of our article. We are glad that you found the article interesting and our methods to be robust and the data well described. You are correct, our method is very similar to distributed peer review (DPR) and we should have included citations and mentions of this in our literature review. Its use in astronomy, and especially by the NSF are particularly interesting, as are the articles which cite its drawbacks. Thank you for improving the paper with mention of this. We have incorporated the mention of our use of DPR throughout the paper.

 We have now expanded the introduction to speak about distributed peer review, and throughout conclusions in the article have also made reference to distributed peer review, although we did not initially use DPR, and were instead adopting a crowd-sourcing approach, and then combined the two methods. These changes have been made especially to the Introduction, where we have incorporated your suggested references, as well as others not mentioned

 In terms of your specific comments, for Figure 2d, the wording has now been changed to reflect the reality that the community review method was expected to be scalable. The use of language was through the assumption of a null hypothesis and was innappropriate. 

 We have also changed the Figure legend of Figure 4c, as pointed out to be reflective of the figure. This was a copy-editing error. The use of the word ' enrichment' has also been specified for Figure 4d.

 Your final point, on removing team composition questions is very interesting especially when thinking about double anonymisation of reviewers. We hope, based on the data of the PCAs which separated the questions by their power and difference to other questions (Figure 3b), that this was not a big influence on the final scores therefore would agree with your own assumption that this wouldn't effect the rankings greatly. Unfortunately although your hypothesis for the inclusion of a further figure to analyse this is sound, our team would prefer to keep the paper in its current state in terms of new hypotheses, but think this is an excellent question and in future publications will ask this very question if given the chance.

 Overall your review has identified some important literature and potential issues, as well as a new hypothesis and we are very grateful for it.

 Thank you for taking the time to analyse our article in the way you have, you've enlightened us specifically on the astronomy field's use of DPR and shown how there is precedent for such methods, with the example of the National Science Foundation's pilot study. Hopefully there will be further attempts with such large organisations in the future, perhaps with safeguarding measures to counteract any collusion, as well as controls studies for grant review with existing techniques.
